# *Profilin1* E117G is a moderate risk factor for amyotrophic lateral sclerosis

**DOI:** 10.1136/jnnp-2013-306761

**Published:** 2013-12-05

**Authors:** Pietro Fratta, James Charnock, Toby Collins, Anny Devoy, Robin Howard, Andrea Malaspina, Richard Orrell, Katie Sidle, Jan Clarke, Maryam Shoai, Ching-hua Lu, John Hardy, Vincent Plagnol, Elizabeth M C Fisher

**Affiliations:** 1 MRC Centre for Neuromuscular Diseases, UCL Institute of Neurology, London, UK; 2 Department of Neurodegenerative Diseases, UCL Institute of Neurology, London, UK; 3 National Hospital for Neurology and Neurosurgery, London, UK; 4 Centre for Neuroscience & Trauma, Blizard Institute, Barts and The London School of Medicine and Dentistry, Queen Mary University of London, London, UK; 5 Department of Molecular Neuroscience, UCL Institute of Neurology, London, UK; 6 Sobell Department of Motor Neuroscience and Movement Disorders, UCL Institute of Neurology, London, UK; 7 UCL Genetics Institute, University College London, London, UK

**Keywords:** ALS, Neurogenetics

## Abstract

**Background:**

Amyotrophic lateral sclerosis (ALS) and frontotemporal dementia (FTD) are progressive neurodegenerative disorders that share significant clinical, pathological and genetic overlap and are considered to represent different ends of a common disease spectrum. Mutations in *Profilin1* have recently been described as a rare cause of familial ALS. The *PFN1* E117G missense variant has been described in familial and sporadic cases, and also found in controls, casting doubt on its pathogenicity. Interpretation of such variants represents a significant clinical-genetics challenge.

**Objective and results:**

Here, we combine a screen of a new cohort of 383 ALS patients with multiple-sequence datasets to refine estimates of the ALS and FTD risk associated with *PFN1* E117G. Together, our cohorts add up to 5118 ALS and FTD cases and 13 089 controls. We estimate a frequency of E117G of 0.11% in controls and 0.25% in cases. Estimated odds after population stratification is 2.44 (95% CI 1.048 to ∞, Mantel-Haenszel test p=0.036).

**Conclusions:**

Our results show an association between E117G and ALS, with a moderate effect size.

## Introduction

Amyotrophic lateral sclerosis (ALS) is a devastating neurodegenerative disorder in which the progressive loss of motor neurons causes weakness and paralysis leading to death typically 3–5 years after onset.^1^ Around 5–10% of ALS cases are familial, which has allowed the identification of a handful of mutant genes that cause classical dominant adult-onset ALS, and are occasionally found in sporadic patients with ALS, who report no family history for the disease.^2^ Frontotemporal dementia (FTD) co-occurs in 5–10% of patients with ALS and the finding that a number of mutations can cause either disorder, and that ALS and FTD postmortem pathology observations share numerous similarities, has led to these disorders being considered part of the same disease spectrum.^3^

Recently, Wu *et al*^4^ have identified missense mutations in Profilin 1 (*PFN1*), a three-exon gene encoding a protein involved in actin filament formation, as causative of ALS. With the exception of the E117G mutation, all mutant *PFN1* alleles were found in familial ALS cases. One successive study identified a novel missense mutation, while a number of screening studies have failed to identify mutations, suggesting these are a rare cause for ALS. By contrast, the E117G mutation was identified in control and familial cases, and was also found at a lower frequency in controls in other populations, thus casting a doubt on the significance of this variant.^5–11^

Here we report the screening of a UK population for *PFN1* mutations. We identify cases carrying the E117G variant and analyse the frequency of this variant in 4700 UK controls. We then meta-analyse all the published and publicly available data for this variant, to conclude this may represent a rare risk factor for ALS.

## Methods

### Study population

Three hundred and eighty-three sporadic and familial ALS cases, from specialist motor neuron disease clinics in London, UK, (UCL Partners motor neuron disease clinics), provided written informed consent. The familial cases were 23 and represent 6% of the cohort. DNA was extracted from whole blood.

### Sequencing analysis

PFN1 exons 1, 2 and 3 were subjected to PCR amplification by REDtaq Polymerase (Sigma), using the primers and PCR conditions as described in Wu *et al.*^4^ Reaction mixtures were made up in accordance with the manufacturer's instructions. The PCR product was purified from the amplification mixture using microCLEAN PCR clean-up kit (Microzone), and the repeat region of interest was sequenced using ABI Prism BigDye Terminator Cycle Sequencing Ready Reaction kit V.1.1. 15 µL sequencing reactions comprised of 5–20 ng of DNA template, 1 µL BigDye mix, 5 µL Better Buffer (Microzone), 5 pmol of the forward and reverse sequencing primer (same as PCR primers), 1 µL betaine and were made up with an appropriate volume of nuclease-free water. Cycling parameters for sequencing were set to 300 s at 95°C, 30 s denaturing at 96°C, 15 s annealing at 55°C, 240 s extension at 60°C and repeated for 35 cycles. Sequences were precipitated in ethanol, EDTA and sodium acetate solution and further washed in 70% ethanol. Sequences were determined on a 3730xl DNA Analyser capillary sequencer (Applied Biosciences) and analysed with Geneious V.6.1 sequencing software (Biomatters).

### Search of public databases and published data

We searched for published sequencing data on *PFN1* in ALS, FTD and control cases from prevalently Caucasian cohorts, updated to 10 May 2013. In particular, we received from the UK10K, (http://www.uk10k.org, a large sequencing study of UK individuals with conditions not related to ALS/FTD) the allele frequency for E117G in 4732 exome sequences.

### Statistical methods

Association p values were based on Fisher exact tests using the allele count data. Stratified population analysis was performed using the stratified Mantel–Haenszel test. All tests were performed using the R statistical software.

## Results

### Sequence screening of PFN1 in a UK ALS cohort

Screening of the three *PFN1* exons in our samples revealed no novel variant. We identified two ALS samples carrying the dinucleotide substitution leading to the previously described E117G coding change. Both patients had no ALS family history. The first patient was a male with bulbar onset ALS, age was 65 years at onset, and a very slowly progressive form of disease. The second patient was a male with limb onset ALS, age was 54 years at onset, and a disease duration of 3 years. Both patients were negative for screening of C9orf72 expansions and mutations in TARDBP, FUS and VCP.

### Meta-analysis of PFN1 E117G variant

To estimate the frequency of the E117G alleles in controls, we combined several publicly available cohorts of exome-sequenced individuals, whose conditions do not overlap with the ALS-FTD spectrum. The largest control cohorts are: NHLBI Exome-sequencing dataset (4300 European–American samples), and the UK10K dataset (4732 UK samples).

We then collected all the published and publicly available data on the E117G *PFN1* variant in ALS and FTD. Combining all cohorts, we obtained information on 5118 cases and 13 089 controls of prevalently European descent.

We initially performed our statistical analysis using the original discovery set from Wu *et al*,^4^ with the following modifications to the control group: (A) we excluded the 1K Genome Project data due to low coverage and potential shortfall in detecting rare variants; (B) we excluded the African–American samples from the Exome Variant Server, since the E117G mutation appears to be specific to European-descent populations; (C) we updated the European-descent samples as on June 2013. This analysis shows an OR=3.71 (Fisher exact test p=0.098) (table 1).

**Table 1:** Frequency of *PFNI* E117G variant

Population	ALS	E117G	FTD	E117G	TOTAL cases	E117G	CONTROL	E117G	p Value	OR (one-tailed 95% CI)	Reference
Original discovery set (after exclusion of African samples in NHLBI dataset, and removal of 1000 genomes cohort)											
North American*	1090	3			1090	3	1089	1			4
European American							4300	3			NHLBI exome sequencing project
Total					1090	3	5389	4	0.098	3.71	
Validation datasets											
UK*	383	2			383	2	4732	8			UK10K (http://www.uk10k.org)
US/Nordic/German*	672	1	16	0	688	1	972	0			7
French	46	0	119	0	165	0					8
Italian*	1168	1	203	0	1371	1	1132	0			9
Canadian	94	0			94	0					5
Australian	825	3			825	3					11
Belgian*	174	0	328	3	502	3	864	3			6
Combined analysis											
Total (all samples, not stratified)	4452	10	666	3	5118	13	13 089	15	0.036	2.22 (1.1 to ∞)	
Total* (stratified Mantel–Haenszel test)					4024	10	8777	12	0.038	2.44 (1.048 to ∞)	

*Indicates case-control datasets used for the Mantel–Haenszel test.

ALS, amyotrophic lateral sclerosis; FTD, frontotemporal dementia.

We then analysed all available datasets which included cases and controls (total of 4024 cases and 8777 controls) and took into account population stratification by using a Mantel–Haenszel test. This analysis showed a significant difference between groups (p=0.036) and an OR of 2.22 (1.1 to ∞ 95% one-tailed CI) (table 1).

By combining all available data, also from non-case-control studies, the variant was present in 13 out of 5118 cases and 15 out of 13 089 control samples conferring similar results to the Mantel–Haenszel test, and showing an OR=2.22 (95% CI 1.1 to ∞ Fisher exact test p=0.036) (table 1).

## Discussion

*PFN1* mutations have been reported to be a rare cause of familial ALS.^4^ ^7^ The *PFN1* E117G variant has been also found in sporadic ALS patients, but its presence in numerous control individuals has raised concerns about its role in disease.^4–11^ With the recent advances in DNA sequencing technologies, information on one patient's whole exome or multiple candidate genes is quickly obtainable—in this context, the interpretation of variants, such as *PFN1* E117G, becomes an important clinical-genetics challenge.

In a new screen of 383 UK patients with ALS, we found two E117G carriers. No novel variant was found. In order to gain insight as to how to interpret the role of this variant, we performed an analysis on all publicly available data for the E117G variant, and we find consistent evidence for the association between E117G and ALS andFTD (p=0.036), with an estimated OR of 2.44 (95% CI 1.048 to ∞ p=0.038).

Wu *et al*^4^ performed functional studies on five *PFN1* mutations, including the E117G variant, demonstrating an effect on protein aggregation, actin binding and axon outgrowth. The E117G variant generally showed intermediate results between the wild-type and the other mutations, reaching significant differences from the wild-type in protein aggregate formation and in increased levels in insoluble protein fractions. Axon outgrowth inhibition was observed, but did not reach statistical significance, and actin binding appeared to be normal.^4^ In summary, functional characterisation of the E117G variant shows lesser changes than clear *PFN1* pathogenic mutations, but supports a possible role as risk factor for ALS.

The possibility of multiple mutations contributing to ALS in one individual,^12^ and the role of rare variants in complex diseases such as ALS and FTD, remains to be elucidated and will require very large cohorts in order to obtain sufficiently convincing results. Recently, Geschwind and collaborators have shown that a rare variant in the *MAPT* gene, where mutations are known to be causative for FTD, can act as a risk factor for FTD and Alzheimer's disease.^13^ Similarly, we here suggest that a rare variant in *PFN1*, a gene in which mutations are known to be causative for ALS, can act as a risk factor for disease.
